# Exploring associations between skin, the dermal microbiome, and ultraviolet radiation: advancing possibilities for next-generation sunscreens

**DOI:** 10.3389/frmbi.2023.1102315

**Published:** 2023-06-23

**Authors:** Matthew L. Smith, Catherine A. O’Neill, Mark R. Dickinson, Bhaven Chavan, Andrew J. McBain

**Affiliations:** ^1^ Division of Musculoskeletal and Dermatological Sciences, School of Biological Sciences, Faculty of Biology Medicine and Health, The University of Manchester, Manchester, United Kingdom; ^2^ Photon Science Institute and Department of Physics and Astronomy, The University of Manchester, Manchester, United Kingdom; ^3^ Personal Care, Croda Europe Ltd, Snaith, United Kingdom; ^4^ Division of Pharmacy and Optometry, School of Health Sciences, Faculty of Biology, Medicine and Health, The University of Manchester, Manchester, United Kingdom

**Keywords:** skin microbiome, sunscreen, ultraviolet radiation, sunlight, microbial selection, skincare

## Abstract

Recent studies have provided strong evidence of a functional link between the microbiota of the skin and overall host health. While sunscreens offer protection against acute and chronic dermatological damage by reflecting, absorbing and scattering ultraviolet radiation, their impact on the skin microbiota is poorly understood. The use of sunscreens may affect the skin microbiota directly or indirectly through mechanisms associated with UV protection, and conversely, the microbiota could mediate or alleviate UV-induced skin damage. Here we consider opportunities for the development of improved sunscreens including formulas that work in tandem with skin commensal microorganisms or which minimise direct effects on the skin microbiota.

## Introduction

Ultraviolet radiation (UVR) from sunlight breaching the atmosphere and interacting with exposed skin can pose a significant health risk due to its directly mutagenic and broad-spectrum tissue damaging properties (Orazio et al., 2013). UVR exposure has been causally linked to conditions such as erythema, cancers of the skin and degenerative aging (Matsumura, 2004; Orazio et al., 2013). A range of topically applied UVR-blocking formulas have been developed, which offer photoprotection for the skin, reducing sun damage (Diffey and Grice, 1997). Whilst the development of sunscreens has contributed to the protection of skin from UVR, there has been little research into the effects of sunscreens on the skin microbiome. Since the microbiome of the skin contributes significantly to health, we propose that the design of sunscreens should consider possible effects on commensal microorganisms.

**Figure 1:**
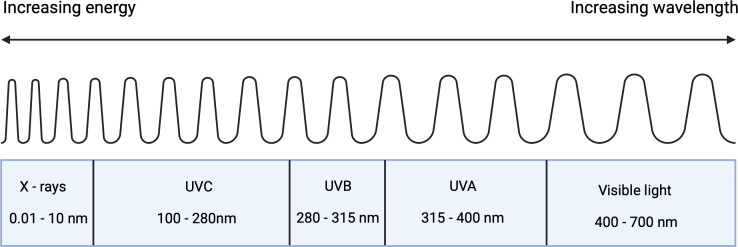
Components of ultraviolet radiation in the perspective of the electromagnetic spectrum (Created with BioRender.com).

UVR consists of a spectrum of various wavelengths, each of which interacts with skin in distinct ways. The three main sub-divisions of UV light are UVA (315-400nm), UVB (280-315 nm) and UVC (100-280 nm) (**Figure 1**) (El-Nouby Adam, 2011). Since photon energy scales inversely with wavelength, UVC would be expected to pose the largest threat to the skin. However, this threat is almost completely eliminated by absorption in the atmosphere. In contrast, UVB is partially absorbed by the atmosphere resulting in a global average of around 8% transmission (El-Nouby Adam, 2011). The atmosphere absorbs considerably less UVA such that solar light reaching the surface of the Earth contains an approximate ratio of 20:1 UVA to UVB. Whilst the dose of UVB interacting with the skin is lower, the higher photonic energies mean that there is still a significant health risk (Kollias et al., 2011). This risk from UVA was often overlooked in early suncare research since data at the time suggested that UVB alone was responsible for UVR-induced skin damage. Subsequent studies demonstrated the damage induced from both UVA and UVB exposure. While UVB is often more strongly associated with immediate and direct DNA damage within the epidermis, UVA may damage deeper dermal layers (Matsumura, 2004; Battie et al., 2014). This has driven the development of sunscreens that protect against UVA and UVB.

Sunscreens originate in the early 1920s, with the discovery that discrete wavelengths of solar UVR are the cause of sunburn (Pathak et al., 1969). This resulted in the rapid development of organic and inorganic UVR blockers – molecules that could be used in topical emulsions applied to the outer layer of the epidermis (stratum corneum) thus enabling photoprotection. P-aminobenzoic acid (PABA) is an example of a key active ingredient used in early sunscreens, although it has since been found to irritate the skin in some cases (Morabito et al., 2011). Research in the field has continued, resulting in the introduction of active pharmaceutical ingredients (APIs) with better UV protection properties, and contemporary sunscreen formulations often contain mixtures of organic, inorganic UV blockers along with and stabilising agents. The efficacy of such products is often tested in human volunteers with the use of methodologies from the International Organisation for Standardisation (ISO), particularly ISO24444:2019 and ISO 24442:2011 which are used to measure UVB and UVA protection, respectively (Zou et al., 2022).

A century of dermatological research has provided the insight needed to develop sunscreen products with increased efficacy. We propose that this should be combined with the expansion of knowledge regarding the skin microbiome, which is largely due to advances in DNA sequencing technology in the form of next-generation sequencing (NGS) (Grogan et al., 2019). Skin microbiome research supports the role of skin commensal bacteria in the skin health and disease. One of many well-documented examples of such interactions being the correlation in *Staphylococcus aureus* in atopic dermatitis (AD) (Byrd et al., 2018) based primarily on DNA sequencing data from AD patients (Byrd et al., 2018). With this in mind, understanding how sunscreens impact the viability, structure and activities of the skin microbiome may play an important role in developing improved sunscreens.

The development of next-generation sunscreens should therefore consider the complex interactions facilitated by commensal microorganisms with human skin. This narrative review, therefore, considers current evidence that might underpin the development of sunscreen products that may work synergistically with natural UVR protection/mediation mechanisms associated with skin commensal microorganisms.

## Sunscreens

Developing an effective sunscreen must balance the need to protect from UVA and UVB, whilst also satisfying consumer preferences (Morabito et al., 2011). This often results in sunscreen formulations being a stable mix of organic or inorganic components, each of which contributes to the absorbance, scattering and reflection of UVR.

For sunscreen products to maintain their consumer value, they must offer photoprotection against acute and chronic UVR exposure, be cost-effective, maintain photostability when exposed to sunlight and they must additionally undergo rigorous testing to ensure their safety when applied to exposed skin (Forestier, 2008). The impacts of sunscreens on the skin microbiome are a likely addition to modern consumer interests.

A common framework for the effectiveness of commercial sunscreens is their sun protective factor (SPF). This is calculated from the minimal dose of sunlight required to cause erythema after applying the product, mostly relating to UVB exposure (Schalka and Reis, 2011). SPF is a widely used tool for assessing the effectiveness of sunscreens but falls short in its lack of consideration of UVA protection. To overcome this, Walgreen Boots Alliance developed a UVA star rating system in 2008. This also considers UVA protection, with a higher star rating representing a higher percentage of UVA absorption compared to UVB (Wang et al., 2008).

### Inorganic UVR blockers

The inclusion of the photoactive nanoparticle (10-60nm diameter) titanium dioxide (TiO_2_) has been a key contributor to the advancement of sunscreens. When used within a formulation the titanium dioxide particles may aggregate which further enhances the capacity for UVR protection, increasing SPF (Morabito et al., 2011). Due to small particle size, TiO_2_ additionally protects against short-wavelength UVR (UVB) exposure (Schneider and Lim, 2019). TiO_2_ has a high refractive index, allowing it to reflect and scatter UVR. TiO_2_ is also a semiconductor with a large gap between energy valances, a feature best described by solid band theory, with a large band gap of 3.1 eV resulting in strong absorption below 400 nm. The incident UVR can therefore lead to electrons being excited into the conduction band leaving behind holes in the valance band (Yang et al., 2004; Feng et al., 2013). Subsequent recombining of these electrons and holes leads to energy release that could then lead to the production of highly oxidising free radicals, potentially leading to the formation of ROS that are damaging to proteins, lipids and DNA (Serpone et al., 2007; Smijs and Pavel, 2011). This can be reduced through the addition of a specialised coating that can decrease its reactivity, thereby eliminating the damaging effects of photoactivation (Jang et al., 2016).

Zinc oxide (ZnO) is another commonly used sunscreen component. ZnO works in a comparable way to TiO_2_ although it can be distinguished through its greater particle size, allowing it to interact with longer UVA wavelengths, while also offering some additional protection to UVB (Smijs and Pavel, 2011; Schneider and Lim, 2019). It is for this reason that the two inorganic compounds are often used in conjunction, offering protection across a broader spectrum of UVR than when used alone. Like its titanium-based counterpart, ZnO may also be formulated with a specialised coating to decrease its photo-reactivity. Common examples of nanoparticle coatings include silicon dioxide (SiO_2_) and aluminium oxide (Al_2_O_3_) which increase the efficiency of UVR blocking, while also markedly decreasing the overall risks associated with photoactivation of the products (Morabito et al., 2011; Smijs and Pavel, 2011; Jang et al., 2016).

### Organic UVR blockers

Organic sunscreen components, commonly referred to as chemical UVR blockers, are classified based on their ability to absorb wavelengths of either the UVA or UVB bands while lacking the properties to either scatter or reflect UVR. Organic blockers are therefore considerably less versatile than their inorganic counterparts and as a result, multiple types must be used to protect from both UVR components (Serpone et al., 2007). Despite the proliferation of their use as sunscreens, reliance on organic UVR blockers has decreased as studies continue to show their shortfalls when compared to inorganic alternatives.

This sole dependence on the absorption of UVR is a large contributor to the susceptibility of organic UV blockers to photodegradation via photolysis, and their ability to generate harmful free radicals. For this reason, there are stringently regulated caps on the concentrations of each type of blocker used within cosmetic sunscreens (Giokas et al., 2007; Serpone et al., 2007). Similar to the use of coatings for inorganic blockers, photo stabilisers may be included within formulations to overcome these negative consequences (Chaudhuri et al., 2006). One such example is diethylhexyl syringylidene malonate (DESM) which improves the photostability of the organic UVA absorber avobenzone, reducing photodegradation which would otherwise lead to ROS production (Chaudhuri et al., 2006).

Organic UV blockers generally have aromatic molecular structures. PABA is an example of such a molecule and it was used abundantly in early sunscreens. Exposure to UVB will result in its absorption by an electron-releasing group, leading to the subsequent delocalisation of electron(s) towards an electron-accepting group. This is termed aromatic electron delocalisation and is an energy-dependent reaction which defines the way PABA can absorb UV light. This is a process seen consistently in many other types of organic blockers. Hydrogen bonding additionally has a role to play in the chemistry behind these molecules, particularly significant in the behaviour of the benzophenones. Here, the presence of internal hydrogen bonding can decrease the energy requirements of electron delocalisation (Shaath, 2010; Shaath, 2016).

As research into organic UV blockers continues, so does the understanding of the complications present through their wide-scale use. Whilst some organic filter detriments are shared with their inorganic counterparts, there are unique issues which may arise with the use of organic blockers, which may deter their use in future. One example of this is their tendency to induce photo-irritant or photosensitising reactions, first observed with the use of PABA in first-generation sunscreens (Morabito et al., 2011). Such considerations may also extend to environmental toxicity (Giokas et al., 2007; Silvia Díaz-Cruz et al., 2008).

## The skin as a microbial habitat

**Figure 2:**
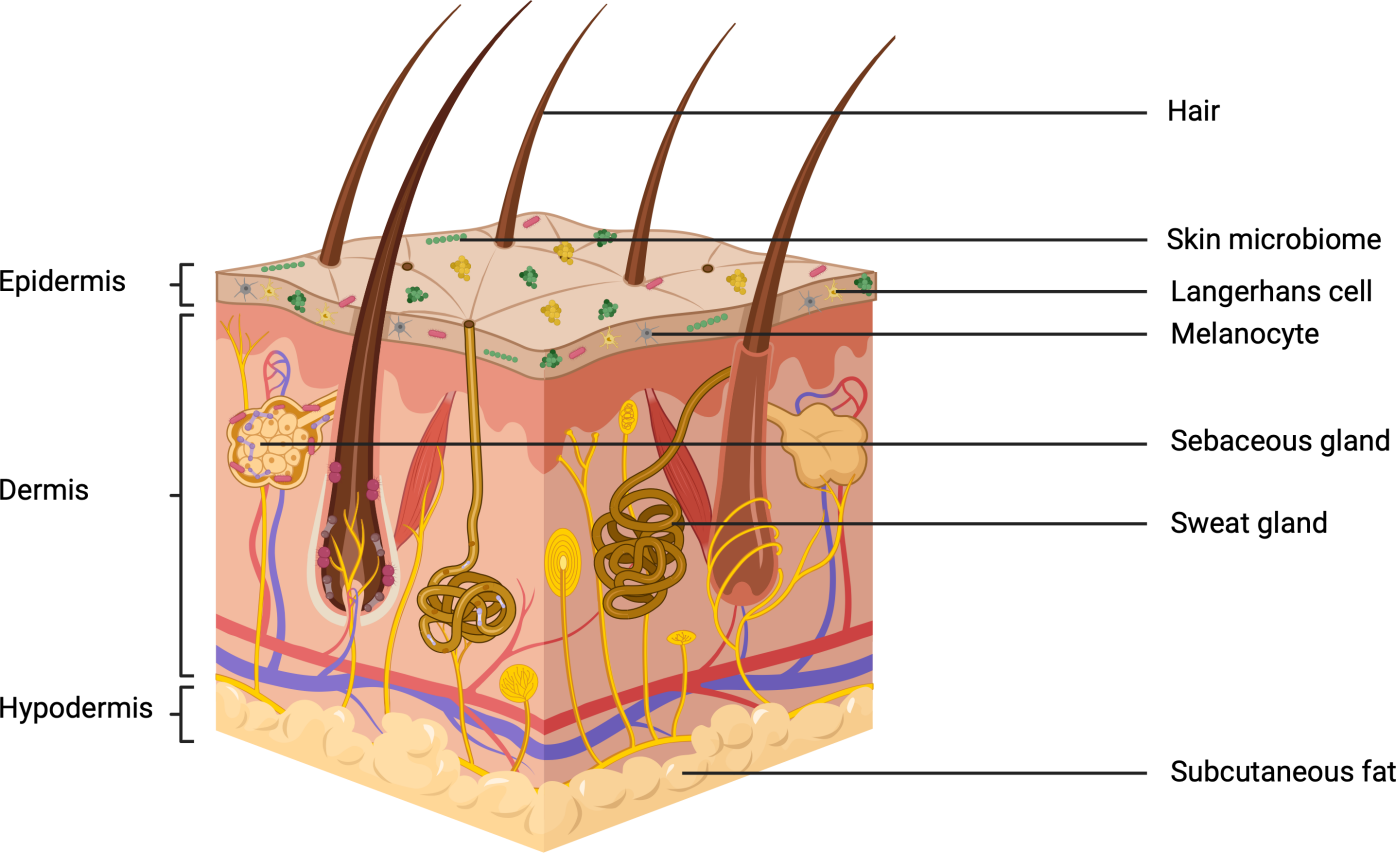
The three subdivisions of skin: the epidermis, dermis and hypodermis. including the relevant appendages and microbiological colonization (Created with BioRender.com).

For reviews on skin structure and function, the reader is referred to Kolarsick and Kolarsick et al, 2011 and Arda and Göksügür, 2014 (Kolarsick et al., 2011; Arda et al., 2014). A focused overview of skin structure is provided in the current review. Skin is composed of three main layers – the epidermis, dermis and hypodermis (**Figure 2**). Together, these form a complex organ that is rich in diverse microbial colonisation. Here trillions of bacteria, fungi, viruses and mites, belonging to over 1000 distinct species reside. This is a dynamic community, comprising both resident and transient microbes the composition of which may alter in response to environmental and biological stimuli, with potentially beneficial or pathogenic implications (Segre et al., 2010; Kong and Segre, 2012; Grice, 2014). Studies suggest that microorganisms, or their extracellular products, can permeate below the epidermal basement membrane and into the lower dermis (Nakatsuji et al., 2013). Skin is not a uniform structure across different anatomical sites, these differing physiologies contribute to the diversity seen within the skin microbiome. This is mostly the result of anatomical differences in the distribution and abundance of accessory structures such as hair follicles, sweat glands and sebaceous glands. This generates a spatial distribution of conditions that can be described as sebaceous, moist or dry - each of which selects for a unique microbial community (Roth and James, 1988; Grice et al., 2009). The complexity of skin as a microbial habitat is further increased through its regular exposure to the UVR in sunlight, the intensity of which often changes due to environmental factors.

### Sebaceous sites

Sebaceous skin is characterised by a high abundance of sebaceous glands. These are responsible for the secretion of lipid-rich sebum which coats the outer epidermis and hair (Greene et al., 1970). Sebum is composed of cell debris (due to the holocrine nature of its production), triglycerides, wax esters and squalene. Other substances may also be present such as fatty acids, often produced through the bacterial breakdown of triglycerides (Thody and Shuster, 1989; Lovászi et al., 2017). Sebum composition is similar across the forehead, chest, back and face (Greene et al., 1970) – all of which are areas of note when considering the application of sunscreens.

The presence of sebum on the skin initially generates a barrier for oxygen transfer, thus potentially selecting for organisms which can grow anaerobically (Leyden et al., 1975; Grice and Segre, 2011). Furthermore, free fatty acids within sebum have been found to exhibit antimicrobial properties. Palmitic and stearic acids commonly occur in sebum and can select against bacteria such as *Pseudomonas aeruginosa* and *Staphylococcus aureus* (Ivanova et al., 2017). Despite these antimicrobial properties, some bacteria have been shown to produce lipases which liberate free fatty acids in sebum, aiding in the microorganism’s ability to adhere to the skin, thereby promoting survivability (Brüggemann et al., 2004). *Cutibacterium acnes* (formerly *Propionibacterium acnes*) is well adapted to sebaceous areas (Brüggemann et al., 2004). *C. acnes* is an aerotolerant anaerobe and can grow in sebum (Brüggemann et al., 2004). The presence of sebaceous glands across the skin is, therefore, a factor that can select for unique skin microbiome compositions, by imposing selective pressures on microbial communities. As a result, the overall microbial diversity in sebaceous areas is reduced compared to that in moist or dry anatomical locations.

### Moist sites

The secretion of sweat onto the skin is a key factor in the development of moist skin. Eccrine sweat glands have an influential role in thermoregulation, secreting sweat across most areas of the skin. The sweat produced here mostly consists of salt and electrolytes, contributing to skin acidification – a factor that often selects against microbial growth (Grice and Segre, 2011). Sweat also contributes to the expression of antimicrobial peptides (AMPs), which may further modulate microbial selection at these sites (Wang et al., 2016). Apocrine sweat glands have a more selective distribution across the skin, commonly being found at the armpit, nipple and genitoanal areas. Apocrine sweat is often associated with bacterial colonisation. Initially, it is a cloudy fluid that contains odourless steroids. However, oxidation of these compounds, by *Corynebacteria*, results in the formation of steroids that cause malodour (Decréau et al., 2003). Sweat can also contain traces of urea, which has been proposed as a potential nitrogen source for staphylococci, offering one explanation as to how they have been so successful in colonising this niche (Grice and Segre, 2011).

Despite selective pressures such as the presence of salts and skin acidification, moist body sites may exhibit high microbial diversity when compared to sebaceous sites. Microbes that prefer humid environments are often found to thrive here, including many *Staphylococcus*, *Micrococcus* and *Corynebacterium* species (Leyden et al., 1981; Byrd et al., 2018).

### Dry sites

Areas of skin such as the forearm, buttock and part of the hand, are considered to be dry environments in terms of microbial colonisation. Dry sites experience the greatest diversity in skin microbiome composition, mostly due to imposing less selective pressures than the other dermatological environments (Grice and Segre, 2011). Utilising molecular techniques, one study reported that gram-negative organisms are present on the dry skin sites of the forearms, despite otherwise being rarely found within the skin microbiome (Gao et al., 2007). However, some organisms within these samples, such as *Pseudomonas*, have been suggested to be transient, with their presence being less common and having a greater correlation to disease than health (Gao et al., 2007).

Phyla such as *Corynebacteria, Cutibacteria, Staphylococcus* and *Acinetobacter* have often been found in skin microbiome samples at dry sites, leading to the assumption that they contribute to the resident microbiome. Despite this, the skin microbiome is highly diversified between individuals, resulting in these common phyla representing only a small proportion of the communities of microorganisms seen across the skin (Gao et al., 2007).

## Photobiology of the skin

The impact of UVR on the skin relies on the presence of chromophores, the photosensitive molecular sites responsible for UVR absorption. These include sites on DNA, amino acids, urocanic acids and other cellular metabolites, which are capable of absorbing the photon energies associated with UVR. This results in either damage that is mediated either directly or indirectly, the latter attributable to the formation of reactive oxygen species (ROS) (Young, 1997; Watson et al., 2014). Such damage often results in the formation of cyclopyrimidine dimers in cellular DNA. If formed in tumour suppressor genes in the absence of successful DNA repair, there may be an increased risk of developing skin cancer (de Gruijl and Rebel, 2008). Chromophores associated with proteins may enter a short-lived excited state after exposure to UVR. This may directly change their molecular structure, potentially causing immediate damage. Protein chromophores may also enter a longer-lived excited state, resulting in the formation of more deleterious ROS, which may additionally cause further damage to the proteins themselves and surrounding tissues. The photosensitive nature of proteins is determined by their amino acid primary structure. Cysteine, tryptophan, histidine and tyrosine have been identified as amino acids particularly vulnerable to UVB. Additionally, tryptophan, tyrosine and cystine (disulphide bonded cysteine) have also shown some vulnerability to UVA-induced damage. Proteins containing higher quantities of these amino acids, particularly if they are on the surface of their quaternary structure, are therefore more likely to be susceptible to UVR absorption, increasing their ability to behave as chromophores (Pattison et al., 2012; Watson et al., 2014).

### The effects of UVR on skin physiology

#### Negative effects

**Figure 3:**
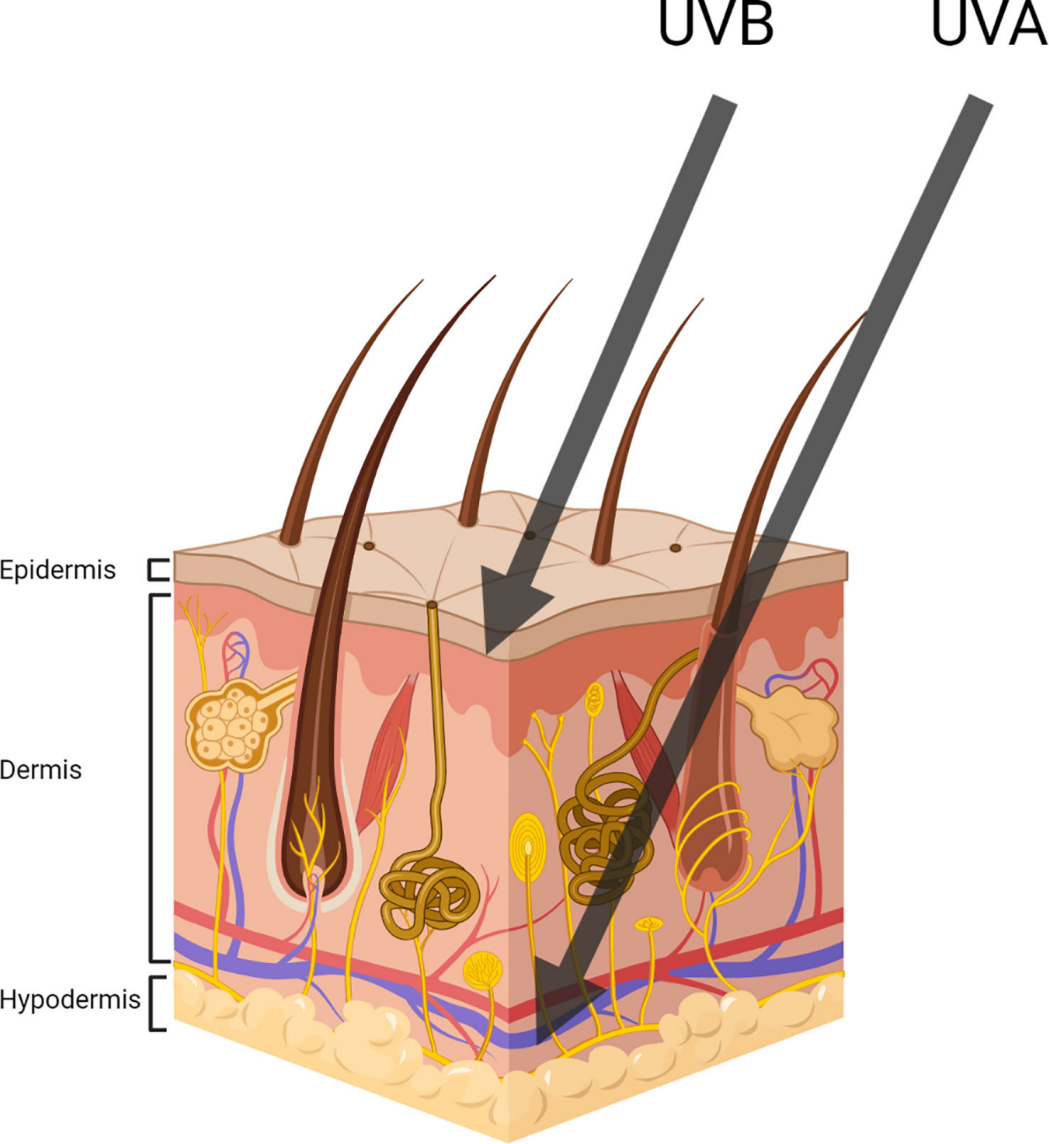
Representing UVA and UVB penetration throughout the three main subdivisions of skin (Created with BioRender.com).

Despite particular photoadaptations having evolved in skin to mitigate sun damage, UVR induces both acute and chronic effects such as mutagenesis, sunburn, degenerative ageing, atrophy and many more deleterious effects, which can compromise health (Matsumura, 2004; Orazio et al., 2013). Both UVA and UVB are key contributors to potential skin damage. Due to their different wavelengths and associated photon energies, each is responsible for distinct types of damage. Notably, the damage caused by UVA is enhanced due to its higher quantities in sunlight (compared to UVB) and its ability to penetrate deeper into the skin (**Figure 3**). UVB on the other hand is more mutagenic, a result of its increased photon energy (Battie et al., 2014).

One example of an acute and often immediate implication of sun exposure is sunburn. UVB interacting with keratinocytes often results in their apoptosis, leading to the formation of sunburn cells (Halliday, 2005). Sun-damaged cells are characterised by their round shape, eosinophilic cytoplasm, condensed nucleus and their development leads to symptoms such as redness, pain, swelling, peeling and blisters (Bernerd and Asselineau, 2008). In contrast to the immediate impacts of UVB exposure, photoaging is a consequence of prolonged, primarily UVA exposure. Photoaging results in the accelerated development of wrinkling, thickening of the skin, dyspigmentation, and decreased elasticity and that in more serious cases may lead to cutaneous malignancies (Gonzaga, 2009). Photoaging is primarily caused through the interaction of UVA with the dermis, contributing to the degradation of extracellular matrix proteins such as collagen and elastin. This effect is cumulative and increases with additional UVR exposure (Gonzaga, 2009).

Photoimmunosuppression is a biological consequence of Langerhans cells being exposed to UVR. This leads to a reduced dendric cell function at low UVR exposures and additional changes to their morphologies at higher exposures. A UVR-mediated depletion in Langerhans cell function results in a decreased T-cell mediated response to foreign antigens, contributing to the immunosuppressive effect seen in skin exposed to sunlight (Schwarz et al., 2000; Novakovic et al., 2001). A clinical ramification here involves the potential for infections to arise in immunosuppressed regions of the skin. One example is the subsequent reactivation of herpes simplex virus infections, in which case UVR may induce cold sores (Rooney et al., 1991). The development of infection is often otherwise mediated through the production of AMPs in sunlight-exposed skin. Immunosuppression is also a significant contributor to the development of skin cancer, once again highlighting the potential consequences of prolonged exposure to UVR (Schwarz, 2002).

One of the most problematic interactions of UVR is its genotoxic impact on exposed skin, inducing mutations which may potentially lead to the development of tumours. One key example of this is the UVR signature mutation of cytosine to thymidine base substitution at dipyrimidine sites. The consequences of this, in the absence of successful DNA repair, could include squamous cell carcinoma, basal cell carcinoma or melanoma (Brash, 2014). However, the damage caused by DNA lesions is often offset through the development of sunburn cells where the damage is so great that the cells apoptose (Bernerd and Asselineau, 2008). In addition to other types of direct DNA damage, UVR produces ROS. One example of this is the photoinduced production of hydrogen peroxide, facilitated by the spontaneous catalysis of anions by the enzyme superoxide dismutase in UV-irradiated melanocytes. Compounds such as hydrogen peroxide can subsequently lead to oxidative damage to DNA, proteins and lipids, all of which are vital to the integrity and function of the skin (Song et al., 2009). Oxidising photoproducts are generated on a large scale, with studies suggesting that each skin cell produces 50-100 photoproducts each second while exposed to direct sunlight. Fortunately, this is often negated by natural repair pathways and is further protected against through the application of sunscreens (Barnard et al., 2018).

#### Positive effects of UV

UVR can also have beneficial impacts on the skin. Exposure to moderate solar radiation, particularly UVB, is the primary inducer of vitamin D synthesis (Hakim et al., 2016). 7-dehydrocholesterol is an early precursor to vitamin D and can be photoisomerised after it absorbs UVB, forming provitamin D_3_. A heat isomerisation reaction then follows this to produce vitamin D, which facilitates the uptake of vital minerals such as calcium, magnesium and phosphate (Webb, 2006). Vitamin D is also known to have profound effects on the immune system (Maruotti and Cantatore, 2010). This exemplifies the need for moderate levels of UVR to maintain health.

UVR can be used therapeutically. An example of phototherapy is in the treatment of psoriasis. This is one of the most common inflammatory conditions of the skin, currently affecting roughly 2% of individuals worldwide (Tsuruta and Imafuku, 2021). The aberrant immune system in this disease causes an imbalance in keratinocyte proliferation and shedding resulting in an increased level of keratinocyte proliferation in the basal layer. At the same time, shedding of squames at the top surface of the skin is reduced leading to the scaly plaques that are characteristic of this condition (Greb et al., 2016). Phototherapy utilises the immunosuppressive action of UVB radiation to dampen the immune system, thereby suppressing keratinocyte proliferation and reducing inflammation (Hönigsmann, 2001).

## The skin microbiome

Bacteria numerically dominate the skin microbiota. Fungi, although often present, have proportionally smaller communities with a lower species diversity than their bacterial counterparts. Abundant fungal colonisers include the genus *Malassezia*, which may be detected across the arms and core body sites. The feet have been associated with greater fungal diversity with *Malassezia* in addition to other fungi such as *Aspergillus* and *Rhodotorula* (Byrd et al., 2018) with corynebacteria, cutibacteria, acinetobacter and staphylococci reportedly being the most commonly occurring bacterial genera (Gao et al., 2007; Grice and Segre, 2011).

The relationship between microorganisms and their hosts may be loosely described as being either mutualistic, commensal or parasitic. This is determined by the benefits or consequences received by either the microorganism or its host and is a factor that can change in response to altering conditions (Schommer and Gallo, 2013). In some instances, a sudden change to microbial community structure (sometimes termed dysbiosis) may result in a shift from communities being mutualistic towards pathogenicity. This has been associated with conditions such as atopic dermatitis, acne and psoriasis (Gao et al., 2008; Kong et al., 2012; Fitz-Gibbon et al., 2013). More research into this relationship is needed to determine whether changes in microbial communities are causal or if they instead occur because of an already changing physiology.

The term pathobiont is often used to describe symbiotic microorganisms with the potential for a pathogenic role, where situational changes increase the benefit of pathogenesis to that species. *Staphylococcus epidermidis* is an example of a pathobiont commonly found in the skin microbiome. This is a key resident species of the skin microbiome and yet it also commonly occurs in infections associated with in-dwelling devices such as catheters (Oliveira et al., 2018). The interaction of pathobionts with the immune system is a principal factor in moderating their behaviour (Patra et al., 2020). Shifts in microbial behaviour and community structure have been linked to several extraneous factors, some of which include clothing choices, hygiene, antibiotic use, occupation, UVR exposure and cosmetic use (Grice and Segre, 2011). Understanding how common skincare practices, such as the use of sunscreens, impact the behaviour of pathobionts is therefore of particular importance.

### The functions of the skin microbiome

Microbial communities on the skin, whether resident or transient, may contribute to the maintenance of skin health. This can be as simple as forming a physical barrier to prevent pathogen invasion but, in many cases, involves more complex and intricate mechanisms such as contributing to the barrier function and maintaining adaptive immunity (Flowers and Grice, 2020; Ito et al., 2021). For example, *S. epidermidis* has been reported to downregulate the proliferation of *S. aureus*, by triggering the enhanced expression of AMPs by keratinocytes (Lee et al., 2019; Brown and Horswill, 2020). Additionally*, S. epidermidis* is involved in the early development of adaptive immunity – specifically in the mucosal-associated invariant T cell priming (Brown and Horswill, 2020). Furthermore, studies show that *S. epidermidis* releases 6-N-hydroxyaminopurine on UVR-exposed skin. This inhibits DNA polymerase activity and thus provides the skin with a protective effect against tumour development (Nakatsuji et al., 2018).

*S. epidermidis*, alongside other coagulase-negative *staphylococci*, is reportedly less abundant in infants who develop atopic dermatitis (AD) (Kennedy et al., 2017). An increase in *S. epidermidis* and *Staphylococcus hominis* growth has additionally been correlated with clinical improvements of AD in a further study (Olesen et al., 2021). *Staphylococcus cohnii* is reported to modulate host anti-inflammatory pathways to protect against the development of AD. The mechanism may involve bacterially induced upregulation of the glucocorticoid production in keratinocytes, reducing the inflammation of local tissues (Ito et al., 2021). These examples suggest a role for the skin microbiome in modulating disease.

Despite the pathogenic tendencies of *S. aureus*, studies have demonstrated its ability to contribute to skin defence against UVB exposure (Ron-Doitch et al., 2021). The eDNA component of its extracellular matrix is suggested to be a key contributor to preventing UVB- induced skin damage. This is probably because of a subsequent activation of the Nrf2–Keap1 pathway which is known to be crucial in cell defence (Ron-Doitch et al., 2021).

Particular strains of *C. acnes* have been associated with acne vulgaris pathogenicity, often arising opportunistically due to the conditions generated from the presence of the disorder (Dréno et al., 2018). Despite this direct link to pathogenesis, *C. acnes* is also reported to have positive effects on the surrounding microbiome and on the skin, often exhibiting antagonistic relationships with *Streptococcus pyogenes*, *S epidermidis and Pseudomonas* species; organisms which if left unchecked may become pathogenic (Byrd et al., 2018; Platsidaki and Dessinioti, 2018).

These examples demonstrate the crucial roles of the diverse microbial communities on skin which contribute to health. Furthermore, these examples suggest an additional level of complexity in the roles of these communities, in which even species believed to be pathogens show the ability to positively impact their surroundings.

### The impacts of UVR on the skin microbiome

The skin microbiome is regularly exposed to UVR. Research indicates that UVR exposure can cause shifts in skin microbiome composition, with UVA and UVB each resulting in different observable effects (Burns et al., 2019). This study however had a moderate sample size and substantial variation between individuals was observed. Despite this it demonstrates impact of sunlight on the skin microbiome. Exposure to UVB has additionally been shown to cause an increase in gut microbiome diversity which was found by analysing participants’ fecal microbiome compositions using 16S rRNA (Bosman et al., 2019) thus furthering the evidence sunlight exposure modulated the human microbiome of the skin.

It has been reported that much like skin cells, bacteria respond differently to the UVA and UVB components of UVR (Burns et al., 2019). An additional study that assessed impact of UVR on *P. aeruginosa* concluded that both UVA and UVB contribute to bacterial inactivation (Oppezzo, 2012). This study provides some insight into the vulnerability of specific microorganisms to UVR. Another study compared the responses of *P. aeruginosa* with those of *Escherichia coli* under the same conditions. Although having a significant impact on *P aeruginosa* viability, UVA had little to no observable effect on *E. coli* (Fernández and Pizarro, 1996).

The effects of UVR on the skin microbiome could occur through direct and indirect processes. UVR poses an immediate threat to mammalian or microbial cells. At the same time, UVR also alters the microbial habitat of the exposed skin, which could induce further changes to the skin microbiome. One indirect mechanism involves the increased expression of AMPs by the skin in response to UVR (Patra et al.; Patra et al., 2018). Some species within the skin microbiome demonstrate resistance to UVR. This is observed for *Micrococcus luteus* which utilises carotenoid pigments and a high endonuclease activity to resist the otherwise bactericidal effects of UVR (Shiota and Nakayama, 1997; Mohana et al., 2013).

Despite the impacts of UVR on the skin microbiome being currently understudied, it is clear that the consequences of exposure can vary between species (Fernández and Pizarro, 1996). This introduces the possibility that UVR exposure can select for organisms, therefore shaping microbial community structure. This, alongside the potential for sunscreens to mitigate such effects by limiting UVR exposure, may be of particular significance in the engineering of next-generation sunscreen products.

### Does the skin microbiome modulate the dermal response to UV irradiation?

Despite having their cellular responses to UVR exposure, microorganisms could be key in mediating how UVR affects the skin. The response of the microbiome to UVB exposure was investigated by Patra et al. in 2019 with the use of germ-free and specific pathogen-free mice. After UVB irradiation the germ-free mice were found to exhibit greater immunosuppression than the specific pathogen-free i.e. microbially colonised mice. This suggests a role for the skin microbiome in reducing the immunosuppressive consequences of UVB exposure (Patra et al., 2019). Recent research has also suggested that the presence of the skin microbiome to be crucial in the maintenance of immune system-related genes, such as those that code for TNF and IL-6 cytokines (Heidari et al., 2020).

### Microbiological defences against UVR

Microbial UVR defences have not only been driven by selective pressure but additionally through the innate ability of UVR to damage DNA, increasing the mutational rate. It is therefore no surprise that microbial species exhibit a vast array of natural adaptations to protect against UVR (Kong and Segre, 2012; Nakatsuji et al., 2018).

An interesting mechanism of UVB resistance has been observed in *S. epidermidis* whereby an electrogenic fermentation-dependent reaction generated electrons which deactivate ROS which may otherwise lead to cellular damage (Balasubramaniam et al., 2020). This does however demonstrates a potential avenue for the defence of the skin microbiome against UVB.

Melanogenesis is a protective response by the skin in minimising damage from incoming solar radiation and is interestingly an adaption that can also be seen in certain microorganisms. Pigments are produced by various skin bacteria including members of the genus *Malassezia*. Other microbial species such as *Streptomyces glaucescens* and have also been shown to produce photoprotective pigments (Oliveira et al., 2018).

The prevalence of UVR defence mechanisms across such a broad range of microbial species demonstrates the threat that UVR poses to the skin microbiome and therefore highlights the need for sunscreens that can provide some form of protection. Furthermore, the presence of UVR protection pathways in particular skin microbiome constituents provides promise for the development of sunscreens in the future. If sunscreens could enhance the UVR protection strategies of naturally occurring pathways found within the skin microbiome, they would likely provide an elevated level of UVR protection. Additionally, with the use of probiotics and prebiotics increasing, a product that naturally encourages the growth of putatively beneficial microorganisms may offer additional benefits to consumers. However, it is important to consider the complex balance of commensal and pathogenic bacteria in this microbiome. For example, clonal expansion of the *Malassezia* might enhance UVR protection but species such as *Malassezia furfur* has been associated with dandruff, highlighting the need for further research (Patra et al., 2020).

### The impacts of sunscreens on the skin microbiome

The effect of commercial sunscreens on the skin microbiome has not been extensively investigated. Research into the effect of specific sunscreen components on the microbiome may be used to make assumptions about the consequences of their use. One example of this is an early study reporting bactericidal effects of ZnO nanoparticles on of *E. coli*, resulting in cell death at concentrations higher than 1.3 x 10^3^ M (Brayner et al., 2006). A later study elaborated on this, including both gram-negative and gram-positive bacteria reporting that concentrations above 1.6 x 10^2^ M ZnO would increase the permeability of bacterial membranes resulting in bacteriostasis or cell death (Huang et al., 2008).

The overall picture of sunscreen will be elucidated through research that considers the diversity of the microbial communities on the skin, the local chemistry of the surface of the skin and the input of solar UVR. Improved understanding could lead to the development of products in the future that are more considerate of this microbiome. Considering the links being drawn between the skin microbiome and health, sunscreens that protect the microbiome from UVR damage could prove to be next-generation products.

## Conclusion

Sunscreens play an important role in protecting the skin from the deleterious effects of UVR. As research continues to highlight both the importance and complexity of the skin microbiome, questions may be raised about the influence of cosmetic practices. The ability of sunscreens to protect the skin microbiome from UVR damage is one area that would benefit from research. Furthermore, research into increasing the efficacy of sunscreens through building on natural UVR protection that may be conferred by the skin microbiome may play an important role in advancing UVR protection. In addition to highlighting important areas of research in this field, this review has considered the potential that they may have in advancing the development of next-generation sunscreens.

## Author contributions

MS produced the first draft of the manuscript with input from all co-authors. AM produced a revised draft after the first peer review. All authors contributed to the article and approved the submitted version.
